# Rapid identification of pyoverdines of fluorescent *Pseudomonas* spp. by UHPLC-IM-MS

**DOI:** 10.1007/s10534-022-00454-w

**Published:** 2022-10-20

**Authors:** Karoline Rehm, Vera Vollenweider, Rolf Kümmerli, Laurent Bigler

**Affiliations:** 1 Department of Chemistry, University of Zurich, Winterthurerstr. 190, 8057 Zurich, Switzerland; 2 Department of Quantitative Biomedicine, University of Zurich, Winterthurerstr. 190, 8057 Zurich, Switzerland

**Keywords:** Pyoverdine, Isopyoverdine, *Pseudomonas*, UHPLC-MS, Ion mobility, Collision cross sections, Collision induced unfolding

## Abstract

Siderophores are iron-chelating molecules produced by bacteria and other microbes. They are involved with virulence in infections and play key roles in bacterial community assembly and as plant protectants due to their pathogen control properties. Although assays exist to screen whether newly isolated bacteria can produce siderophores, the chemical structures of many of these bio-active molecules remain unidentified due to the lack of rapid analytical procedures. An important group of siderophores are pyoverdines. They consist of a structurally diverse group of chromopeptides, whose amino acid sequence is characteristic for the fluorescent *Pseudomonas* species that secrets them. Although over 60 pyoverdine structures have been described so far, their characterization is cumbersome and several methods (isoelectrofocusing, iron uptake measurement, mass determination) are typically combined as ambiguous results are often achieved by a single method. Those additional experiments consume valuable time and resources and prevent high-throughput analysis. In this work, we present a new pyoverdine characterisation option by recording their collision cross sections (CCS) using trapped ion mobility spectrometry. This can be done simultaneously in combination with UHPLC and high-resolution MS resulting in a rapid identification of pyoverdines. The high specificity of CCS values is presented for 17 pyoverdines secreted by different *Pseudomonas* strains. The pyoverdine mass determination by full scan MS was supported by fragments obtained from broadband collision induced dissociation (bbCID). As iron contaminations in laboratories are not uncommon, CCS values of ferripyoverdines were also evaluated. Thereby, unusual and highly characteristic ion mobility patterns were obtained that are suitable as an alternative identification marker.

**Supplementary Information:**

The online version contains supplementary material available at 10.1007/s10534-022-00454-w.

## Introduction

Secondary metabolites are low-molecular-weight compounds that are not essential for the growth or reproduction of microorganisms but confer a certain ecological benefit. They take on roles as toxins deployed as weapons against competing organisms, metal transporters, quorum sensing molecules for chemical communication, biosurfactants to modulate group motility and many more (Rosenberg and Ron 1999; Demain and Fang 2000; Waters and Bassler 2005; Braud et al. 2009; Cezard et al. 2015). As they display numerous bioactive properties, they can be used as a basis to develop novel antibacterial and antitumor drugs, or immunosuppressants (Demain 1999; Demain and Sanchez 2009; Pita-Grisanti et al. 2022). Moreover, secondary metabolites such as biosurfactants are of interest for industrial applications as they are environmentally more sustainable than their chemical counterparts (Marchant and Banat 2012).

Secondary metabolites include siderophores, molecules with a high binding affinity for iron (> 10^30^ M^−1^). Siderophores have medical, industrial and agricultural applications (Saha et al. 2016). They can deliver antibiotics to cells (Mislin and Schalk 2014) and be used as anticancer drugs (Pita-Grisanti et al. 2022). They detoxify heavy metal contaminated soils (Hesse et al. 2018) and have probiotic activities to protect plants from pathogen infections (Gu et al. 2020a). Furthermore, there is increasing evidence that they play an important role for microbiome community assembly (Gu et al. 2020b; Figueiredo et al. 2022).

As siderophores are specific to the bacterial species that secrete them, a large molecular diversity exists. To date, over 500 chemically distinct compounds have been characterized and new siderophores are discovered yearly (Hider and Kong 2010). Due to this complexity, the chemical structures of pathogen promoting or inhibiting siderophores remain often unknown despite great interest. One class of bioactive and structurally diverse siderophores are pyoverdines (Butaite et al. 2017; Gu et al. 2020a).

**Fig. 1:**
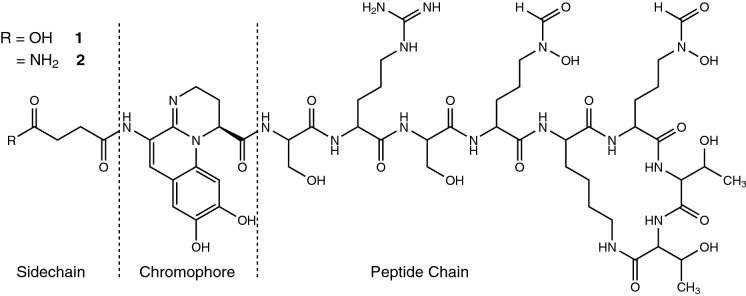
PAO1 with an succinic acid (**1**) or succinic amide (**2**) as an exemplary pyoverdine structure illustrating the fixed pyoverdine framework containing a sidechain, the pyoverdine chromophore and a structurally divers peptide chain

Pyoverdines, such as the one produced by PAO1 (**1** and **2**, see Fig. 1), are iron-chelating chromopeptides consisting of 6–14 amino acids and are produced exclusively by fluorescent *Pseudomonas*. Until today, over 60 pyoverdine types were structurally elucidated (Meyer et al. 2008a). To save time and resources, rapid identification methods for pyoverdines have been developed in which a measured characteristic of an unknown pyoverdine is matched against a library database of known pyoverdines. Such characteristic markers can be obtained for example by isoelectrofocusing (IEF) and by determination of the pyoverdine-mediated iron uptake behaviour of bacteria (Meyer 2000). In most cases, either IEF patterns or iron-uptake experiments deliver unique results that provide a reliable pyoverdine identification.

However, ambiguous findings are sometimes observed. This is due to close structural similarities of different pyoverdines such as the mere replacement or addition of a single amino acid residue (Meyer et al. 2002, 2008b). Membrane receptors for the iron transport cannot distinguish between small differences and matching IEF patterns make a differentiation impossible. Thus, Meyer et al*.* proposed a more advanced identification method using mass spectrometry (MS) (Meyer et al. 2008b). Their study showed that the molecular mass of pyoverdines is another highly specific marker, with only few exceptions where different pyoverdines have an identical mass. Hence, the combination of mass spectrometry with either IEF or iron-uptake experiments are recommended. Alternatively, pyoverdine precursor ions can be fragmented by MS/MS resulting in mainly B and Y″ (Y-ion plus two H atoms) ions. These fragments can be used for a total elucidation of the pyoverdine peptide chain (Budzikiewicz et al. 2007b; Wei and Aristilde 2015; Baune et al. 2017; Rehm et al. 2022). Such a structural elucidation is time-intensive and requires specialized expertise, whereas IEF and iron uptake experiments call for additional equipment that is not always accessible. Therefore, an alternative pyoverdine characterization method, that is rapid, easy, and specific, would be desirable.

One of the latest techniques for characterizing compounds is trapped ion mobility spectrometry (TIMS) by which collision cross sections (CCS) of molecules can be measured. In combination with UHPLC (ultrahigh-pressure liquid chromatography) and MS, a separation in three dimensions can be achieved: Polarity, shape, and mass. The strength of TIMS lies in the separation of isomers and the analysis of complex mixtures. A significant popularity gain is seen in the field of lipidomics, proteomics, isomers as well as positional post-translation modifications on proteins (Fouque and Fernandez-Lima 2019).

Regarding pyoverdine and its iron bond complex, no ion mobility data is published up to date to our knowledge. Furthermore, no data on any covalent metal-peptide complexes could be found either, even though simple peptides have been thoroughly analyzed by IM-MS (Wu et al. 2000; Silveira et al. 2014). CCS values possess a large potential to replace iron uptake and IEF experiments as they depend on the intrinsic volume of an analyte and can be recorded simultaneously during an UHPLC-MS measurement saving time and equipment.

In our study, the application of ion mobility in combination with UHPLC and MS was investigated for the purpose of characterizing and identifying pyoverdines produced by fluorescent *Pseudomonas*. Employing a timsTOF Pro, unique CCS values were collected for pyoverdines produced by 17 different bacterial strains. To enhance the screening of bacterial extracts for pyoverdines, characteristic fragments were generated by broadband collision induced dissociation (bbCID) that could be effortlessly assigned to precursor ions with the help of ion mobility separation even in highly complex samples. The analysis of ferripyoverdines delivered unusual and highly characteristic ion mobility patterns that were shown to be suitable as an alternative identification marker.

## Materials and methods

### Chemicals and reagents

Commercial pyoverdines from *P. fluorescens* (Py SA, CAS RN 8062-00-8, > 90%) and 2,2′-bipyridine (≥ 99%) were obtained from *Sigma Aldrich* (Buchs, SG, Switzerland). Acetonitrile (CH_3_CN), methanol (MeOH) and isopropanol were obtained from *Biosolve* (ULC grade, Valkenswaard, Netherlands) and formic acid from *Fluka* (LC–MS grade, Buchs, SG, Switzerland). Ultrapure water (< 2 ppb TOC) was produced using a Milli-Q® Advantage A10 water purification system (*Merck*, Bedford, MA, USA). Iron(III) chloride hexahydrate of 99 + % purity was bought from *ACROS Organics* (Fair Lawn, NJ, USA). For mass and ion mobility calibration, a 1:1 mixture of ESI-L low concentration tune mix bought from *Agilent* (Santa Clara, CA, USA) and a 10 mM sodium formate solution was prepared. The 10 mM sodium solution contained 1 M NaOH (250 μL) and formic acid (50 μL) in 50% isopropanol (25 mL). For the growth of bacteria, a casamino acid solution (CAA) was used as a nutrient medium. This aqueous solution (1 L) was made from vitamin free casein acid hydrolysate (10.0 g), K_2_HPO_4_·3H_2_O (1.18 g) and MgSO_4_·7H_2_O (0.25 g). The listed ingredients were bought from *Sigma Aldrich* (Buchs, SG, Switzerland).

### Bacterial cultures

All 16 cultures were stored as glycerol stocks at − 80 °C and stemmed from the Rolf Kümmerli strain collection (University of Zurich). The three *P. aeruginosa* strains PAO1, 1–60, and 206-12 produced pyoverdine type 1, 2, and 3, respectively (Smith et al. 2005; Visca et al. 2007). Furthermore, the pyoverdines of 13 *Pseudomonas* environmental isolates were investigated. They were sampled from soil and pond habitats on the campus of the University of Zurich Irchel (47.40° N, 8.54° E; Switzerland) (Butaite et al. 2017) and their pyoverdine structures were elucidated in the work of Rehm et al. (2022). A complete sample ID list is found in Table 1 stating the pyoverdine peptide chain sequences and their masses based on the pyoverdine derivative with a succinic acid side chain (Suc-Py) as recommended by Meyer et al. (2008c).

**Table 1:** Overview of the bacterial isolates and their produced pyoverdines analysed in this study

Sample ID	Peptide chain	Sum formula	Nom. mass (Da)	[*M* + H]^+^ (*m/z*)	[*M* + 2H]^2+^ (*m/z*)
206-12	(Ser-Dab)-FoOHOrn-Gln-Gln-FoOHOrn-Gly	C_48_H_67_N_15_O_20_	1173	1174.47650	587.74216
1–60	Ser-FoOHOrn-Orn-Gly-aThr-Ser-cOHOrn	C_45_H_65_N_13_O_19_	1091	1092.45979	546.73381
3A06	Asp-ε-Lys-OHAsp-Ser-Thr-Ser-Lys-cOHOrn	C_52_H_76_N_14_O_23_	1264	1265.52860	633.26821
3B19	Asp-FoOHOrn-Lys-(Thr-Ala-Ala-Lys-FoOHOrn-Lys)	C_61_H_93_N_17_O_22_	1415	1416.67593	708.84188
3C16	Asp-Ala-Asp-AcOHOrn-Ser-cOHOrn	C_44_H_60_N_11_O_20_	1047	1048.38596	524.69689
3D19	Ala-AcOHOrn-Gly-Gly-Ser-Ala-OHAsp-Thr	C_43_H_57_N_12_O_22_	1122	1123.41799	562.21291
3F12	Lys-AcOHOrn-Gly-Thr-Thr-Gln-Gly-Ser-cOHOrn	C_55_H_82_N_16_O_22_	1318	1319.58678	660.29730
3G07	Asp-FoOHOrn-Lys-(Thr-Ala-Ala-FoOHOrn-Lys)	C_55_H_81_N_15_O_21_	1287	1288.58097	644.79440
PAO1	Ser-Arg-Ser-FoOHOrn-(Lys-FoOHOrn-Thr-Thr)	C_55_H_83_N_17_O_22_	1333	1334.59768	667.80275
Py SA	Ser-Lys-Gly-FoOHOrn-(Lys-FoOHOrn-Ser)	C_49_H_72_N_14_O_19_	1160	1161.51764	581.26273
S3a05	Lys-AcOHOrn-Ala-Gly-aThr-Ser-cOHOrn	C_47_H_69_N_13_O_18_	1103	1104.49618	552.75200
S3a20	ɛ-Lys-OHAsp-Ala-aThr-Ala-cOHOrn	C_42_H_59_N_11_O_17_	989	990.41686	495.71234
S3b09	Asp-ε-Lys-OHAsp-Ser-aThr-Ala-Thr-Lys-cOHOrn	C_56_H_83_N_15_O_24_	1349	1350.58136	675.79459
S3b16	Ala-Lys-Thr-Ser-AcOHOrn-cOHOrn	C_45_H_66_N_12_O_17_	1046	1047.47471	524.24127
S3c13	Ser-Val-OHAsp-Gly-Thr-Ser-cOHOrn	C_43_H_59_N_11_O_20_	1049	1050.40161	525.70472
S3e20	Ser–Lys–Ala–Ser–Ser–AcOHOrn–Ser–Ser–cOHOrn	C_53_H_79_N_15_O_23_	1293	1294.55515	647.78149
S3g01	Ala-AcOHOrn-Ala-Gly-Ser-Ala-OHAsp-Thr	C_46_H_64_N_12_O_22_	1136	1137.43364	569.22073

Py SA refers to the commercially bought pyoverdine reference material. Sum formulas and masses are based on the corresponding pyoverdine derivative with a succinic acid side chain (Suc-Py). Parentheses refer to ring closures. (Unusual amino acids: OHAsp, threo-β-hydroxy-Asp; OHOrn, N^4^-hydroxy-Orn Ac(Fo)OHOrn, N^4^-acetyl-(formyl) OHOrn; cOHOrn, cyclo-OHOrn (3-amino-1-hydroxy-piperidone-2); aThr, allo-Thr)

### Pyoverdine production in liquid cultures

Liquid cultures were grown according to the protocol described in the work of Rehm et al. (2022) which is briefly summarised as follows: bacteria were incubated overnight at 28 °C in lysogeny broth (8 mL) under vigorous shaking (170 rpm). Afterwards, the cultures were centrifuged (7500 rcf, 3 min) and the supernatant was discarded. The remaining pellet was washed twice with 0.8% NaCl (8 mL). 0.8% NaCl was added to the washed pellet until an optical density at 600 nm (OD600) of 1 absorbance unit (AU) was reached. 2 mL of the resuspended culture were grown in CAA (500 mL) supplemented with 250 μM 2,2′-bipyridine (1.25 mL) at 28 °C under vigorous shaking (170 rpm) for 72–120 h. The supernatant of the liquid culture was then filtered through a 0.22 μm filter before being stored at − 20 °C.

### Sample preparation

Sample preparation took place following the protocol published by Rehm et al. (2022). There, a solid phase extraction (SPE) was performed using the Strata-X (1 mL, 30 mg) polymeric reversed phase cartridges from *Phenomenex* (Torrance, CA, USA). In short, the cartridges were washed with 1 mL MeOH followed by equilibration with 1 mL H_2_O. 500 µL supernatant was acidified with 5 µL formic acid and was loaded onto the sorbent. The sorbent was washed with 0.6 mL H_2_O and pyoverdines were eluted using 0.6 mL 30% MeOH in H_2_O containing 0.1% formic acid. The elute was stored at − 20 °C until use.

The pyoverdine reference material purchased from *Sigma Aldrich* was dissolved in 50% MeOH in H_2_O to a concentration of 50 μg/mL.

In order to obtain ferripyoverdine complexes, 10 μL of a freshly prepared FeCl_3_ solution (10 mM in H_2_O) was added to 200 µL of a 5 °C cool pyoverdine extract or reference solution. The mixture was briefly vortexed whereupon a color change from colorless to light brown was observable indicating a completed complexation reaction.

### Chromatographic conditions

Liquid-chromatography was performed on a Vanquish Horizon UHPLC System by *Thermo Fisher* (Waltham, MA, USA) build from a Vanquish binary pump H, a Vanquish split sampler HT and a temperature-controllable Vanquish column compartment. Chromatographic separation was achieved at 25 °C on an ACQUITY UPLC HSS C18 Column (100 Å, 1.8 µm, 2.1 × 100 mm, *Waters*, Milford, USA). Eluent A consisted of H_2_O + 0.1% HCOOH and B of CH_3_CN + 0.1% HCOOH. The following gradient was applied at a constant flowrate of 0.4 mL: (i) 4% B isocratic from 0.0 to 0.5 min; (ii) linear increase to 11% B until 5.25 min; (iii) linear increase to 95% until 7.0 min; (iv) holding 95% B until 10.0 min (vi) back to the starting conditions of 4% B until 10.5 min; (vii) equilibration for 4.5 min until the next run. The injection volume amounted 5 µL.

### Instrumentation

A timsTOF Pro hybrid quadrupole-time-of-flight (QTOF) mass spectrometer employing trapped ion mobility spectrometry (TIMS) produced by *Bruker* (Bremen, Germany) was connected to the Vanquish UHPLC system and was used to acquire mobility and MS/MS data. Ionisation was performed in positive ESI mode. Source parameters were set as followed: End plate offset of 500 V, capillary voltage of 4000 V, nebulizer pressure of 2.7 bar (N_2_), heated dry gas flow of 8.0 L/min with a temperature of 220 °C. Mass and CSS calibration took place using the *Agilent* low concentration tune mix diluted in a sodium formate solution prior to analysis. For additional mass accuracy, a calibration segment was programmed from 0.2 to 0.3 min at every UHPLC run with the help of a 6-port-valve with a 20 µL loop. For data acquisition, the mass spectrometer was operated in TIMS on mode. Based on the recommendations for reporting IM-MS measurements (Gabelica et al. 2019), the relevant TIMS parameters are stated in the following: A ramp time of 250 ms was chosen with an inverse reduced mobility range of 0.55–1.25 1/K_0_. Ion charge control (ICC) was active with a target count of 5 million. The radiofrequency of the TIMS ion funnels was set at 350 Vpp. The Δ6 (ramp start → accumulation exit) voltage was set at 100.0 V. On average, the TIMS cartridge tunnel-in pressure and the tunnel-out pressure amounted 2.6 mbar and 0.8 mbar, respectively. N_2_ of at least 4.5 purity was provided by the university main supply and was used as a drift gas after purification by a HC Big Supelpure HC Hydrocarbon Trap from *Sigma Aldrich* (Buchs, SG, Switzerland). MS/MS spectra were acquired by bbCID with a collision energy of 80.0 eV. For the evaluation and interpretation of the measured results, DataAnalysis 5.2 was used. A one cycle Savitzky Golay algorithm over 10 points (0.005 [V*s/cm^2^]) was applied to smooth ion mobility spectra. A mass deviation up to 5 ppm was tolerated.

## Result and discussion

### Chromatographic separation

The chromatographic conditions were adapted from the publication Rehm et al. (2022) that provides extensive data on all apo-pyoverdines (Py). Here, we transferred the method to a Vanquish Horizon UHPLC system and recorded additional retention times of the detected ferripyoverdines (FePy). All measurements were conducted in triplicate. In Table 2, an overview over all retention times is given. Even though the complexation by mere addition of FeCl_3_ was successful for each sample, ferripyoverdine with a succinic amide side chain (Suca-FePy) of sample 1–60 and the uncyclized ferripyoverdine with a succinic acid side chain (Suc-FePy) of sample 3B19 could not be detected as the concentration of the free apo-pyoverdines was originally close to the detection limit. In all but two cases (pyoverdine with a succinic acid side chain (Suc-Py) of sample 206-12 with a linear structure and pyoverdine with a succinic amide side chain (Suca-Py) of sample 3B19 with a cyclised structure), a significant shift in retention time upon iron complexation could be observed. While the change in retention time is comparable for a pair of Suc-Py and Suca-Py upon complexation, no general applicable rule can be made regarding the overall impact of complexation on the polarity of the compounds. From a 3 min earlier up to a 0.5 min later elution of the iron complex, a broad range of retention time shifts was noticeable. As each pyoverdine has its own specific iron binding sites influencing individually the 3D shape of the complex and the outer chemical groups interacting with the column material, this result was to be expected.

**Table 2:** Average retention times of identified pyoverdines and their corresponding iron complexes of each sample

Sample ID	RT of Apo-Pyoverdines [min]		RT of Ferripyoverdines [min]	
	Suc-Py	Suca-Py	Suc-FePy	Suca-FePy
206-12 cyclized^a^	2.09	–	2.37	–
206-12 linear^b^	2.03	–	2.02	–
1–60	2.31	1.43	0.99	–
3A06	2.74	1.99	2.57	1.82
3B19 cyclized^a^	1.87	1.03	0.91	1.05
3B19 linear^b^	1.74	–	–	–
3D19	4.36	3.62	2.67	2.23
3F12	3.91	3.16	1.52	1.10
3G07 cyclized^a^	2.86	2.17	2.25	1.25
3G07 linear^b^	2.79	–	1.57	–
PAO1	3.84	3.19	3.63	3.00
Py SA	3.11	2.38	2.38	2.09
S3a05	4.13	3.41	1.08	0.93
S3a20	4.26	3.53	3.90	3.36
S3b09	3.24	2.58	3.71	3.02
S3b16	3.77	3.14	2.33	1.90
S3c13	5.70	4.92	3.41	2.61
S3e20	3.70	3.02	1.61	1.15
S3g01	4.98	4.30	2.98	2.33
3C16	Glu-IsoPy		Glu-FeIsoPy	
	3.92		2.20	

^a^“Cyclized” refers to a ring formation within the peptide chain

^b^“Linear” refers to the linear/open version of the molecule. (Suc-Py: pyoverdine with a succinic acid side chain, Suca-Py: pyoverdine with a succinic amide side chain, Glu-IsoPy: isopyoverdine with a glutamic acid side chain, Suc-FePy: ferripyoverdine with a succinic acid side chain, Suca-FePy: ferripyoverdine with a succinic amide side chain, Glu-FeIsoPy: ferriisopyoverdine with a glutamic acid side chain)

**Fig. 2:**
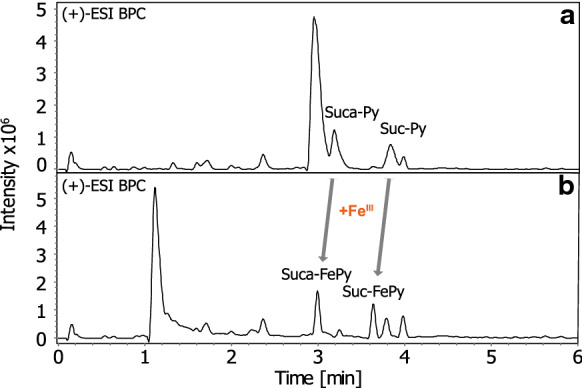
Base peak chromatograms (*m/z* 50–2000) from 0 to 6 min of a PAO1 extract (**a**) and a PAO1 extract spiked with iron (**b**). (Chromatograms were smoothed by a one cycle Savitzky Golay algorithm over 10 points.)

Concerning the peak shapes and intensity, an explementary base peak chromatogram is depicted in Fig. 2. After addition of iron to a PAO1 extract, peak shapes remain sharp with comparable intensities. Compounds that do not bind towards iron retain their retention time whereas the newly formed iron complexes are clearly distinguishable from their apo-forms. All in all, our chromatographic conditions are a robust choice to separate not only apo-pyoverdines but also metal complexes.

### CCS values of pyoverdines

CCS values of pyoverdines from 17 bacterial samples were determined by TIMS-MS. Masses of identified pyoverdines lied between 989 and 1433 Da. Pyoverdine CCS values were derived from their [M + 2H]^2+^ ions due to their high abundance and ranged from 340.9 to 400.7 Å^2^. As multiple kinds of side chains may exist for a single pyoverdine type, this study put a focus on the pyoverdines with a succinic acid (Suc-Py) and a succinic amide side chain (Suca-Py). Moreover, the iso-pyoverdine with a glutamic acid side chain (Glu-IsoPy) produced by the 3C16 strain was included. All CCS values were calculated based on an average of three measurements together with their standard deviation. The mean standard deviation amounted 0.17 Å^2^ (± 0.17 Å^2^) with the highest deviation being 1.08 Å^2^ and the lowest 0.01 Å^2^. This is in great accordance to already published values (0.20 ± 0.15 Å^2^) for the accuracy of the timsTOF Pro (Schroeder et al. 2020). Due to the excellent chromatography and the high instrument resolution, clean ion mobility spectra (figure S1-S19 in the supplementary) were obtained that were not distorted by any isobars. All CCS values are listed in Table 3.

**Table 3:** CCS values of apo-pyoverdines and ferripyoverdines including relative intensities in percent of the measured ion mobility peaks

Sample ID	CSS [Å^2^]												
	Suca-Py	Suc-Py	Suca-FePy					Suc-FePy					
Peak #	1	1	1	2	3	4	5	1	2	3	4	5	6
1–60	351.6	351.6						352.3	359.1	367.6			
	100%	100%						46%	100%	15%			
206-12 cyclized	n.d	358.8	n.d					353.5	366.4	377.1			
	n.d	100%	n.d					100%	19%	5%			
206-12 linear	n.d	365.6	n.d					359.2	368.9	379.5			
	n.d	100%	n.d					100%	74%	9%			
3A06	374.3	371.3	370.3	380.7	386.8			369.2	381.5	386.3			
	100%	100%	100%	84%	56%			93%	100%	62%			
3B19	385.3	390.1	389.5	397.0	402.3	410.1	418.0	378.6	387.8	395.8	398.1	409.1	
	100%	100%	50%	92%	54%	100%	20%	20%	49%	65%	69%	100%	
3B19	n.d	400.7	n.d					n.d					
	n.d	100%	n.d					n.d					
3D19	349.0	350.4	343.7	356.0	375.2			343.0	355.2	369.6			
	100%	100%	66%	55%	100%			36%	28%	100%			
3F12	387.8	388.4	376.1	384.9	391.7	397.9		379.8	388.0	391.7			
	100%	100%	24%	41%	74%	100%		90%	60%	100%			
3G07 cyclized	394.3	382.1	370.5	379.9	387.0	395.6	410.5	366.3	371.8	378.9	386.3	394.9	409.2
	100%	100%	5%	23%	7%	100%	7%	5%	7%	16%	8%	100%	6%
3G07 linear	n.d	376.9	369.5	379.0	385.5	390.7	393.5	365.4	369.3	373.9	379.0	384.1	392.0
	n.d	100%	100%	33%	43%	14%	17%	100%	27%	8%	21%	10%	5%
PAO1	386.1	383.0	374.5	384.3	391.9			374.0	382.8	391.0			
	100%	100%	13%	44%	100%			11%	48%	100%			
Py SA	369.4	366.8	355.3	370.6				354.8	370.1				
	100%	100%	100%	53%				100%	53%				
S3a05	356.8	355.9	350.2	360.7	364.2	367.7		348.6	355.3	363.0			
	100%	100%	100%	20%	22%	16%		100%	12%	29%			
S3a20	341.4	340.9	337.6	343.9				337.9	343.4				
	100%	100%	100%	17%				100%	32%				
S3b09	391.2	389.8	380.3	386.6	390.4	397.1	401.2	379.5	385.4	389.2	397.6		
	100%	100%	100%	49%	24%	32%	13%	100%	53%	31%	47%		
S3b16	363.6	363.4	351.4	355.3				342.2	350.4	354.8			
	100%	100%	100%	74%				60%	100%	58%			
S3c13	344.7	345.0	348.6	352.8	357.6			348.3	352.5				
	100%	100%	27%	81%	100%			28%	100%				
S3e20	381.7	381.2	375.2	381.5				376.1	381.5				
	100%	100%	100%	39%				100%	62%				
S3g01	348.2	349.4	347.4	357.4	360.8	369.3		347.0	355.2	360.9	370.1		
	100%	100%	100%	40%	39%	17%		100%	77%	65%	19%		
	Glu-IsoPy		Glu-FeIsoPy										
3C16	347.1		332.9	347.7	351.9	373.3	376.2						
	100%		7%	20%	100%	9%	9%						

**Fig. 3:**
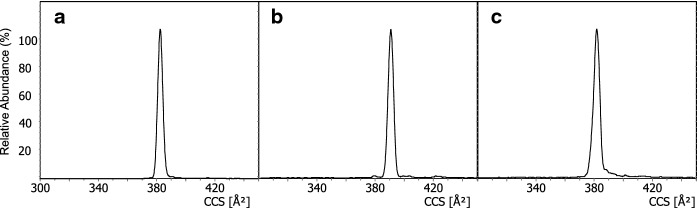
Ion mobility peak shapes of Suc-Py extracted from sample PAO1 (**a**), S3b09 (**b**) and 3G07 (**c**)

All apo-pyoverdines showed a single dominant ion mobility signal. Three Suc-Py examples are shown in Fig. 3. Sharp peaks are gained for cyclopeptides as produced by PAO1 (a) and for linear pyoverdines as found in sample S3b09 (b). Interestingly, all measured cyclo-depsipeptides owned a slightly broader peak shape as in the example of 3G07 (c).

**Fig. 4:**
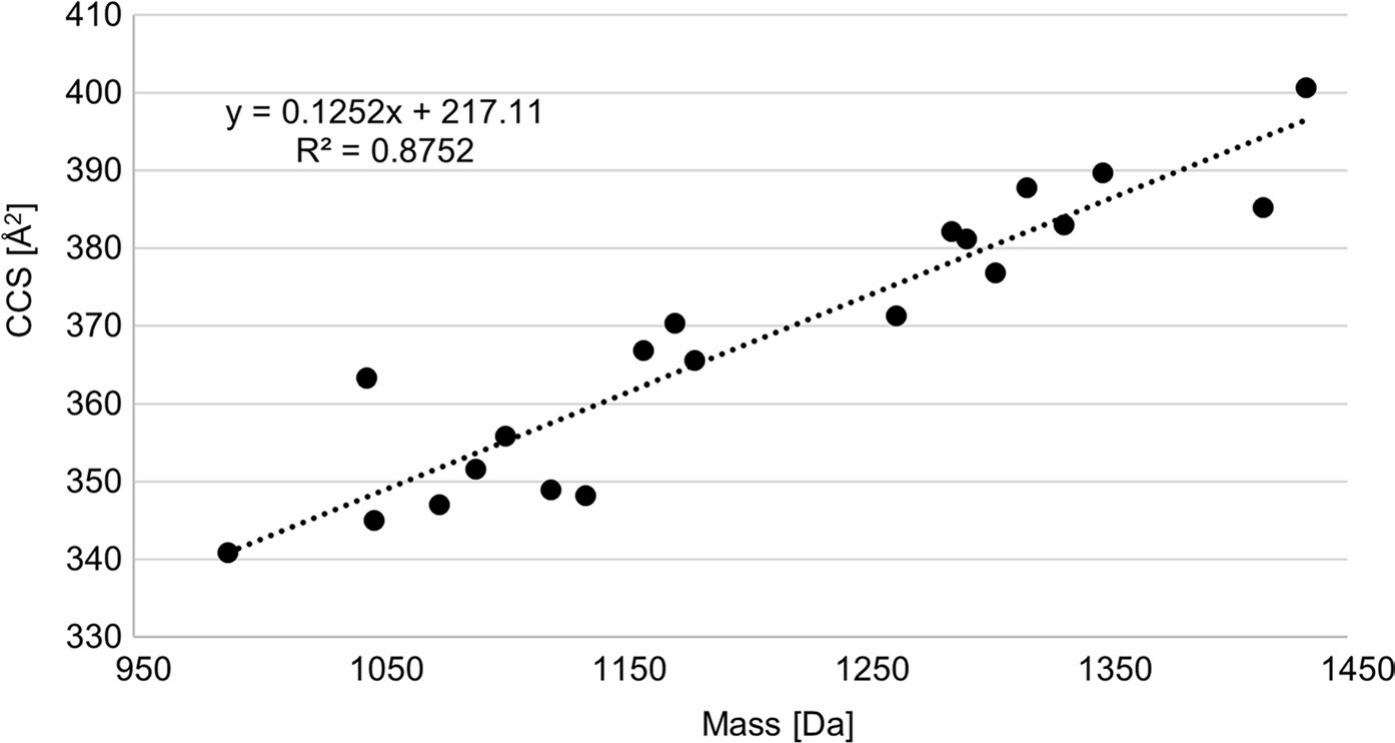
CCS values of Suc-Py compounds plotted against their corresponding nominal mass in Da accompanied by a trendline

When plotting CCS values against their corresponding nominal mass, a clear trendline is visible as illustrated in Fig. 4. The outlier with a small mass of 1046 Da but a large CCS value of 363.4 Å^2^ belongs to the Suc-Py of S3b16 that owns a short but very branched peptide chain. Comparing Suc-Py with their Suca-Py derivatives, the collision cross section of the corresponding Suca-Py is on average by 1 Å^2^ larger. As the CCS of pyoverdines have never been recorded before to the best of our knowledge, no comparison to literature values could be made.

Overall, very distinctive CCS values were obtained even for pyoverdines similar in mass. These CCS values heavily depend on the amino acids appearing in the peptide sequence and are highly individual characteristic markers. If one were to record CCS values of all known pyoverdines, a database could be created to allow a fast matching between measured and theoretical CCS values. In this way, pyoverdines from fluorescent *Pseudomonas* can be identified not only by their unique *m/z* value but can also be confirmed by their corresponding CCS value in a single measurement run. Hence, the potential of ion mobility measurements to effectively replace further iron uptake or IEF experiments has been demonstrated. Moreover, a trendline correlating *m/z* and CCS values can be plotted as seen in Fig. 4. While this trendline is not suitable to confirm the identity of a pyoverdine, it can be used to roughly estimate the expected CCS value of a pyoverdine with a known mass but unknown structure.

### Screening for characteristic pyoverdine fragments: application of bbCID with IMS

The workflow of identifying pyoverdines by mass spectrometry sounds simple: compare the measured pyoverdine mass with reference mass lists of pyoverdines and identify the peptide chain. Yet, finding the correct pyoverdine mass is a challenge as a mixture of various pyoverdine derivatives and possibly other compounds is always obtained after a purification of a liquid bacterial culture. One idea to solve this issue might be to filter the data by ion mobility. More precisely, only compounds could be displayed that are located near the pyoverdine *m/z* to CCS trendline (see Fig. 4). However, this approach is not feasible. In the work of Picache et al., it was shown that molecular classes such as lipids or nucleotides owe characteristic *m/z* to CCS trendlines that partially overlap in some cases (Picache et al. 2019). While they did not analyse pyoverdines, they did collect data on classic underivatized peptides. These classic peptides are encountered in a *m/z* to CCS range that is very similar to pyoverdines. The pyoverdine *m/z* to CCS trendline is therefore not specific enough to filter out only this molecular class.

**Fig. 5:**
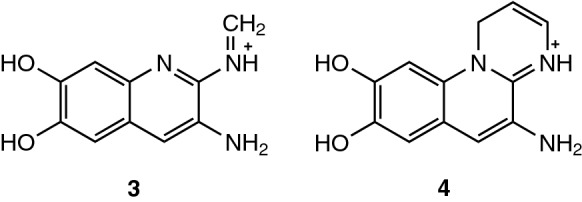
Characteristic fragments for pyoverdine (**3**) and isopyoverdine (**4**)

Hence, an additional fast way to screen for specific pyoverdine signals during the same analysis run is desirable. This can be achieved by all ion fragmentation as conducted in the studies of Wei and Aristilde (2015) or Rehm et al*.* (2022). Pyoverdines and related compounds give characteristic fragments containing the chromophore unit making a rapid identification of precursor candidates possible. The fragments at *m/z* 204.0768 (C_10_H_10_O_2_N_3_^+^) (**3**) and *m/z* 230.0924 (C_12_H_12_N_3_O_2_^+^) (**4**) are encountered for pyoverdine and isopyoverdine, respectively (see Fig. 5, Budzikiewicz et al. 2007a). Afterwards, targeted MS/MS is usually applied in a second experiment. There, single precursors are isolated in order to prove the origin of the characteristic chromophore fragment to the supposed pyoverdine mass. In targeted MS/MS spectra, the side chain as well as the first amino acid unit of the peptide chain can be then easily identified by searching for the first A1 or B1 fragment. In Table 4, the most common A1 and B1 fragments for pyoverdines with a succinic acid side chain are listed as reported by Budzikiewicz et al*.* (2007b).

**Table 4:** Dominant A1 or B1 fragments expected to be found in MS/MS spectra of pyoverdines with a succinic acid side chain depending on the 1st amino acid residue as reported by Budzikiewicz et al*.* (2007b)

1st amino acid of the pyoverdine peptide sequence	Expected fragment [*m/z*]
Ser	417.1405 (A1)
Ala	401.1456 (A1)
ε-Lys	458.2034 (A1)
Lys	486.1983 (B1)
Asp	473.1303 (B1)

**Fig. 6:**
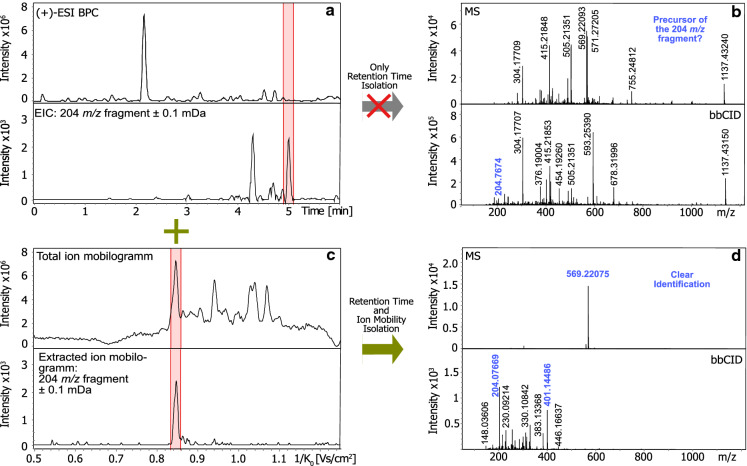
Base peak chromatogram of the bacterial extract S3g01 together with the extracted ion chromatogram of the 204 *m/z* fragment (**a**). Full Scan MS and bbCID spectra obtained after integration of a time range of 4.9–5.1 min (**b**). Total ion mobilogramm and extracted ion mobilogramm of *m/z* 204 in the time range 4.9–5.1 min (**c**). Full Scan MS and bbCID spectra obtained after integration of a time range of 4.9–5.1 min and of a reduced ion mobility range from 0.84–0.86 1/K_0_

Combining all ion fragmentation with TIMS replaces the need of targeted MS/MS experiments. The exemplary bacterial extract of S3g01 is illustrated in Fig. 6. In its base peak chromatogram (a), one dominant signal at 2.2 min is present together with several lower abundance peaks. Extracting the 204 *m/z* fragment of the bbCID measurement results in two clear signals. We obtain complex MS and MS/MS spectra when integrating over the corresponding retention time of one of the peaks (b). An identification of the precursor mass of the characteristic fragment is not possible. With TIMS, we can additionally extract the ion mobility range of the fragment (c). As the precursor ion owns the same CCS value as the characteristic fragment ion, the result is a clean MS and corresponding MS/MS spectrum (d). We can easily identify the 569 *m/z* signal as the precursor. Moreover, we obtain a cleaner MS/MS spectrum where the side chain as well as the very first amino acid of the pyoverdine’s peptide sequence is effortlessly distinguishable. In this example, the A1 fragment (401 *m/z*) containing a succinic acid side chain and an alanine residue was detected.

In all of our analysed samples, pyoverdine fragments could be easily assigned to its precursor mass either due to the excellent chromatographic separation, an abundant pyoverdine concentration or the extraction of the ion mobility trace of the characteristic fragment. Hence, an additional targeted MS/MS scan for pyoverdine confirmation as in former reported methods was not needed. Even though bbCID does not isolate single precursor masses like other untargeted approaches such as parallel accumulation-serial fragmentation (PASEF), it provides an overall higher sensitivity and still allows for a clear precursor identification when combined with ion mobility separation. The danger of a mass not being picked for fragmentation is avoided and several bbCID scans are given across a peak delivering a higher mass accuracy. For the aim of finding compounds belonging to a targeted chemical class, this approach gave the best results and worked for all of our samples.

In our study, a fragmentation energy of 80 eV was ideal to produce characteristic pyoverdine fragments over a broad range of compounds with mass errors under 5 ppm. In Table 5, an exemplary overview for the fragments found for all Suc-Py and Glu-IsoPy compounds is provided. As the side chain has little influence on the pyoverdine fragmentation behaviour, Suca-Py gave identical A1 and B1 fragments with a mass difference of − 0.9840 *m/z.* The 204 *m/z* and 230 *m/z* fragment were detected effortlessly for each pyoverdine and isopyoverdine, respectively. Furthermore, the first amino acid (Ser, Asp, Lys, ε-Lys or Ala) could be successfully identified in all cases with the exception of the pyoverdine of sample 206-12 whose first amino acid, serine, underwent a condensation reaction with the following Dab residue. However, the serine containing A1 fragment of the corresponding uncyclized pyoverdine was easily detected.

**Table 5:** Characteristic fragments as well as A1/B1 fragments detected by bbCID (80 eV) for all Suc-Py and Glu-Py compounds

Sample ID	Measured characteristic fragment *m/z*	Ppm error	Chromophore type	Measured first A/B ion	Ppm error	Fragment type	First amino acid residue	Side chain
206-12 cyclized^a^	204.07678	0.13	Pyoverdine	x				
206-12 linear^b^	204.07682	0.33	Pyoverdine	417.14212	3.94	A1	Ser	Suc
1–60	204.07663	− 0.60	Pyoverdine	417.14235	4.49	A1	Ser	Suc
3A06	204.07691	0.77	Pyoverdine	473.13004	− 0.56	B1	Asp	Suc
3B19 cyclized^a^	204.07655	− 0.99	Pyoverdine	473.13083	1.11	B1	Asp	Suc
3B19 linear^b^	204.07650	− 1.24	Pyoverdine	473.13026	− 0.09	B1	Asp	Suc
3C16	230.09242	− 2.31	Iso-pyoverdine	484.14465	− 3.39	A1 – H_2_O	Ala	Glu
3D19	204.07671	− 0.21	Pyoverdine	401.14556	0.00	A1	Ala	Suc
3F12	204.07651	− 1.19	Pyoverdine	486.19929	1.99	B1	Lys	Suc
3G07 cyclized^a^	204.07665	− 0.50	Pyoverdine	473.12972	− 1.24	B1	Asp	Suc
3G07 linear^b^	204.07645	− 1.48	Pyoverdine	473.12991	− 0.83	B1	Asp	Suc
PAO1	204.07597	− 3.84	Pyoverdine	417.14017	− 0.73	A1	Ser	Suc
Py SA	204.07602	− 3.59	Pyoverdine	417.14035	− 0.30	A1	Ser	Suc
S3a05	204.07609	− 3.25	Pyoverdine	486.19680	− 3.14	B1	Lys	Suc
S3a20	204.07621	− 2.66	Pyoverdine	458.20364	0.50	A1	ε-Lys	Suc
S3b09	204.07670	− 0.26	Pyoverdine	473.13021	− 0.20	B1	Asp	Suc
S3b16	204.07650	− 1.24	Pyoverdine	401.14534	− 0.55	A1	Ala	Suc
S3c13	204.07675	− 0.01	Pyoverdine	417.14034	− 0.32	A1	Ser	Suc
S3e20	204.07634	− 2.02	Pyoverdine	417.14137	2.14	A1	Ser	Suc
S3g01	204.07650	− 1.24	Pyoverdine	401.14485	− 1.77	A1	Ala	Suc

Overall, we proved that bbCID in combination with ion mobility is not only suitable to screen for pyoverdines but is also capable to deliver information on the first amino acid residue of the peptide chain as well as the nature of the side chain in a single measurement run. When identifying pyoverdines based on their mass, further IEF and iron-uptake experiments are recommended as a few cases exist where pyoverdines with a different peptide chain own an identical mass (Meyer et al. 2008b). However, these few pyoverdines differ in their first amino acid, which can be easily distinguished in our bbCID approach. Hence, an unambiguous pyoverdine derivative identification is possible that can be further supported by the comparison of measured and database saved CCS values. In this way, isolated MS/MS fragmentations and time-consuming fragment interpretation can be avoided for already known peptide sequences whereas unknown pyoverdine structures are recognized faster.

### Ferripyoverdines: change in CCS patterns

Iron contaminations are not seldom in laboratories compromising the analysis of the apo-pyoverdine (Baune et al. 2017). Hence, it was decided to also determine the CCS values of ferripyoverdines by the addition of FeCl_3_ to the pyoverdine extracts.

The mass difference between the monoisotopic masses of apo-pyoverdine and ferripyoverdine amounts 52.91146 *m/z* due to the addition of Fe^+3^ and loss of 3 H atoms. Additionally, iron owns a characteristic isotopic pattern with a X − 2 isotope of about 6% making the complex easily recognizable in a MS spectrum. After the addition of iron, the signals of apo-pyoverdines disappeared and new signals for ferripyoverdine appeared. As the iron-containing extract was separated by UHPLC and ferripyoverdines showed different retention times compared to their iron-free form, we assume that true iron complexes and not loose iron adducts were detected. Following injections of iron-free pyoverdine extract resulted in the detection of only the apo-pyoverdine proving that no iron is absorbed by the column material.

**Fig. 7:**
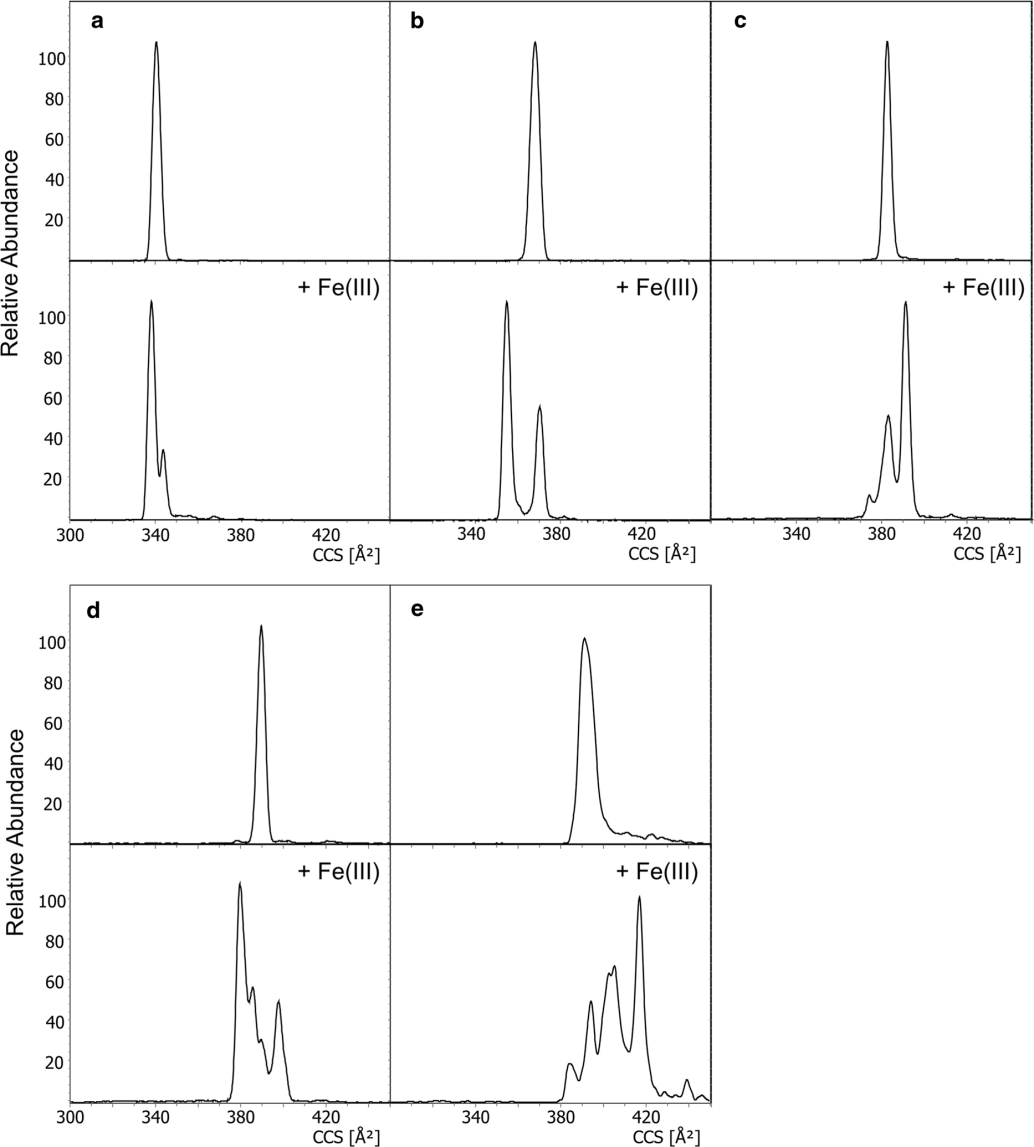
Change in ion mobility pattern upon iron complexation of Suc-Py of S3a20 (**a**), Py SA (**b**), PAO1 (**c**), S3b09 (**d**) and 3B19 (**e**)

Upon evaluation of the CCS values of the ferripyoverdines, a change in ion mobility pattern was observed in comparison to the corresponding apo-pyoverdines. This change was heavily compound dependent and could not be predicted as illustrated in Fig. 7. For example, the Suc-FePy signal of S3a20 and Py SA gave two clear signals (a and b), the Suc-FePy of PAO1 three (c), the Suc-FePy signal of S3b09 four (d) and 3B19 even five signals (e). The intensity ratio of the signals remained the same for each measurement. When determining an average CCS value of the ferripyoverdine and comparing it to the CCS of the corresponding apo-pyoverdine, only a minor shift (− 1.9 ± 7.0 Å^2^) was found. Hence, the ion mobility pattern change is more relevant. All CCS data of the ferripyoverdines including the relative intensities of the ion mobility peaks are listed in Table 3 and can be visually found in figure S1-S19 in the supplementary.

The reason for the appearance of more than one signal could be manifold: Coordination isomerism, helical handedness or partial molecule unfolding. In the case of the pyoverdine PAO1, a detailed conformational analysis in solution by NMR was already published by Wasielewski et al*.* (2008). There, two major conformers of the ferripyoverdine structure have been observed. For one conformer, one possible structural configuration was considered while for the other conformer two different configurations would have fit according to their NOE and dihedral angle restraint energy calculations. Hence, three different 3D shapes were presented which would be in agreement with the three ion mobility peaks found in this study. For the remaining ferripyoverdines, neither conformational data nor reference CCS for comparison could be found.

Taken together, mobility measurements of ferripyoverdines delivered stable and reliable CCS values. Due to the highly specific ion mobility patterns of the complexes, pyoverdine identification based on CCS values can also be conducted in situations where an iron-free environment for analysis cannot be guaranteed. Thereby, the importance of reporting each ion mobility signal must be highlighted.

### Collision induced unfolding of ferripyoverdines: the importance of the Δ6 voltage

Potential collision induced unfolding (CIU) of ferripyoverdines was investigated as comparable ion mobility spectra have to be ensured across platforms. CIU is a method to analyze structural dynamics of molecules by energetical activation. This means that folded and unfolded states of a molecule can be observed. For the timsTOF Pro, this can be achieved by the manipulation of the Δ6 parameter. This parameter defines the transfer voltage to bring ions from the accumulator tunnel into the separation tunnel. In other words, it adjusts the velocity of the ions during the transfer. By increasing the velocities, ions will collide with the drift gas with higher collision energies. Considerable increase of the voltage can lead to “in-TIMS” fragmentation as reported for several small molecule such as rutin and naringin by Schroeder et al*.* (2020). As only few reported studies on larger proteins such as ubiquitin employing CIU exist (Liu et al. 2018), the investigation of the structural dynamics of smaller ferripyoverdines is of particular interest.

**Fig. 8:**
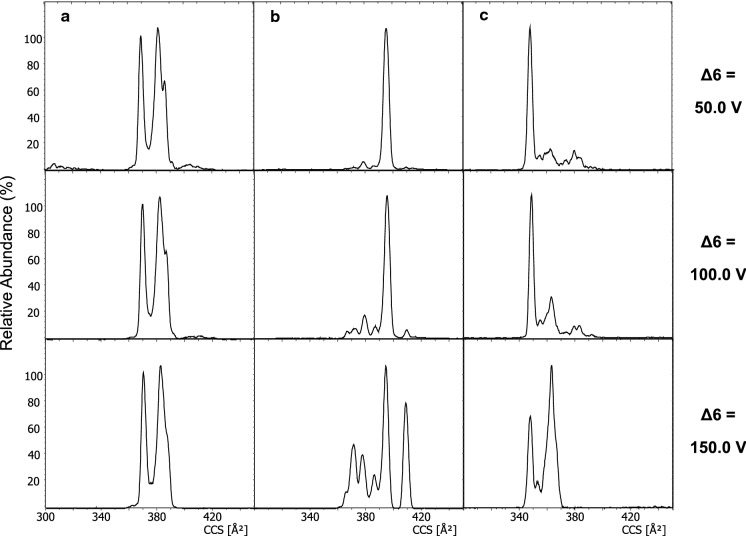
Extracted ion mobilogramms of Suc**-**FePy of 3A06 (**a**), 3G07 (cyclized) (**b**) and S3a05 (**c**) at three different Δ6 voltages (50.0, 100.0 and 150.0)

Ion mobilogramms of the iron complexes (Suc-FePy and Suca-FePy) were recorded at a Δ6 voltage of 50.0 and 150.0 V and compared to the spectra measured at 100.0 V. In general, two categories of ferripyoverdines could be differentiated: Those that were influenced by the transfer voltage and those that were not. In Fig. 8, three of such examples are presented. While the mobility patterns of Suc-FePy of 3G07 (cyclized) (b) and S3a05 (c) start to change heavily upon an increased 150.0 V Δ6 value, the overall peak distribution of Suc-FePy of 3A06 (a) remains the same. The ion mobility behavior of Suc-FePy and their corresponding Suca-FePy were almost identical. This result shows the importance of uniform measurement settings. Only when CCS values and patterns are reported in a detailed fashion with all relevant parameters, results can be repeated and compared. In the end, a Δ6 voltage of 100.0 V was chosen as it suppresses ion mobility artifacts while still ensuring little impacts of CIU on the complexes.

## Conclusion

As the interest in siderophores rises due their versatile application in medical, technical and environmental settings, rapid analytical methods for their identification are necessary. In this study, we presented a new approach to classify pyoverdines secreted by fluorescent *Pseudomonas* by UHPLC-IM-MS in combination with bbCID overcoming the need of work-intensive MS/MS interpretations or further equipment for iron-uptake or isoelectrofocusing studies. Over 17 pyoverdines differing in peptide chain were analysed using a timsTOF Pro. IMS was shown to deliver highly specific CCS values that can be used to characterize pyoverdines replacing the need of other analytical methods. In combination with bbCID, pyoverdine characteristic fragments were generated that could be effortlessly assigned to their precursors. As not all laboratories can ensure an iron-free environment, CCS values of ferripyoverdines were also recorded. It was shown that these iron complexes do not only differ in CCS value but also split into characteristic ion mobility patterns that can be used as a characteristic marker. Thereby, the instrument settings should be reported and kept constants for all future experiments since some complexes are prone towards collision induced unfolding changing the overall appearance of the ion mobility pattern. On this basis, measurements of many more pyoverdines could be conducted in the future to build a library of CCS values to be used as a reference. In this way, research on the field of the biological activity and applications of pyoverdines can be facilitated.

## Supplementary Information

Below is the link to the electronic supplementary material.

Supplementary file1 (PDF 809 KB)
