# Supplementary material for: Rapid identification of pyoverdines of fluorescent Pseudomonas spp. by UHPLC-IM-MS
Source: Biometals. 2022 Oct 20;36(1):19–34. doi: 10.1007/s10534-022-00454-w (PMC9925543; doi:10.1007/s10534-022-00454-w)

Electronic Supplementary

Rapid Identification of Pyoverdines of fluorescent *Pseudomonas* spp. by UHPLC-IM-MS

Karoline Rehm, Vera Vollenweider, Rolf Kümmerli, Laurent Bigler

S1. Ion mobility patterns of Suc-Py (a) and Suc-FePy (b) of 206-12 cyclized and Suc-Py (c) and Suc-FePy (d) of 206-12 linear measured at a Δ6 value of 100.0 eV.

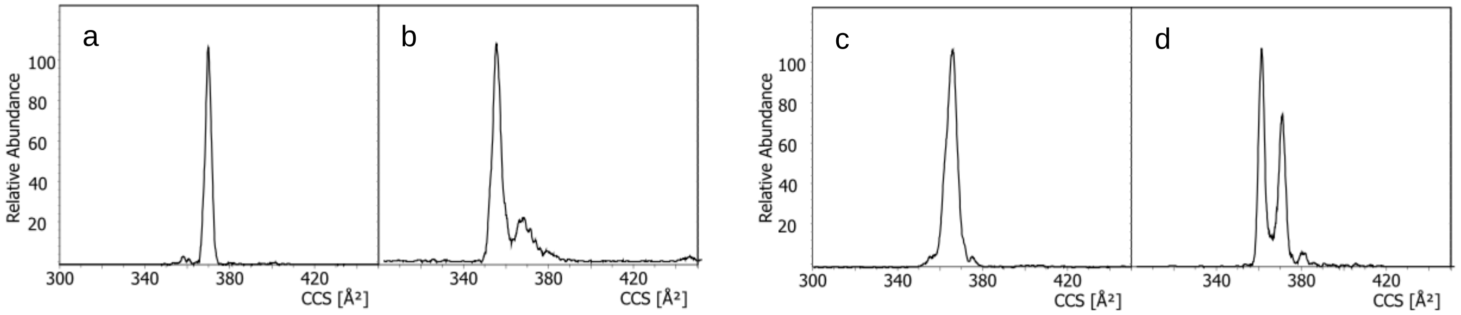

S2. Ion mobility patterns of Suc-Py (a), Suc-FePy (b), Suca-Py (c) and Suca-FePy (d) of 1-60 measured at a Δ6 value of 100.0 eV.

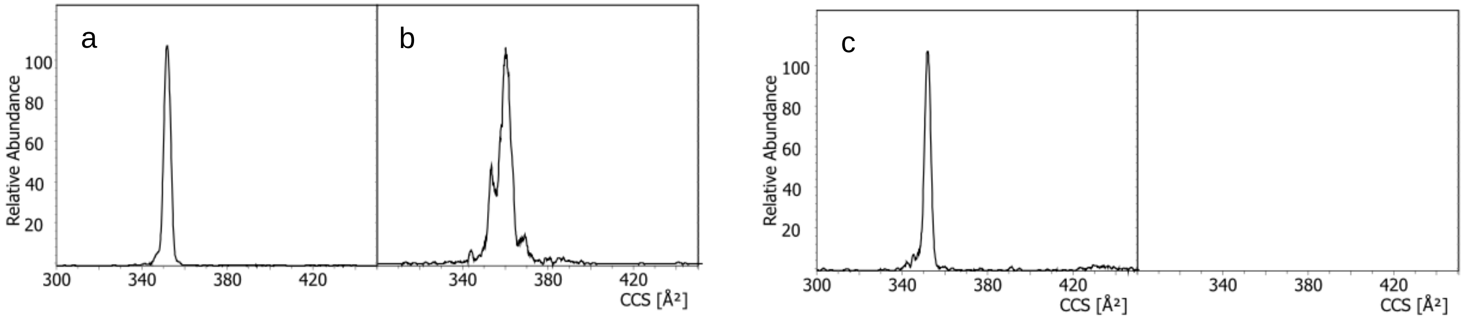

S3. Ion mobility patterns of Suc-Py (a), Suc-FePy (b), Suca-Py (c) and Suca-FePy (d) of 3A06 measured at a Δ6 value of 100.0 eV.

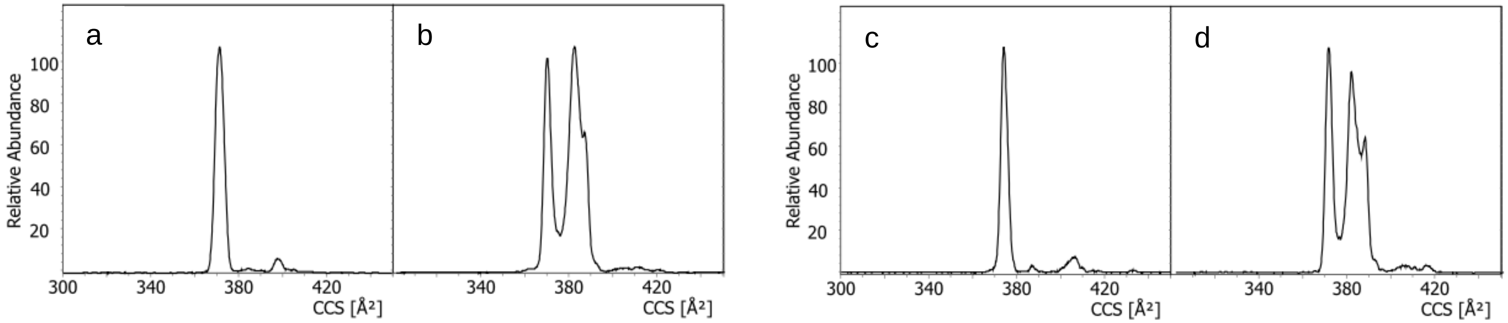

S4. Ion mobility patterns of Suc-Py (a), Suc-FePy (b), Suca-Py (c) and Suca-FePy (d) of 3B19 cyclized measured at a Δ6 value of 100.0 eV.

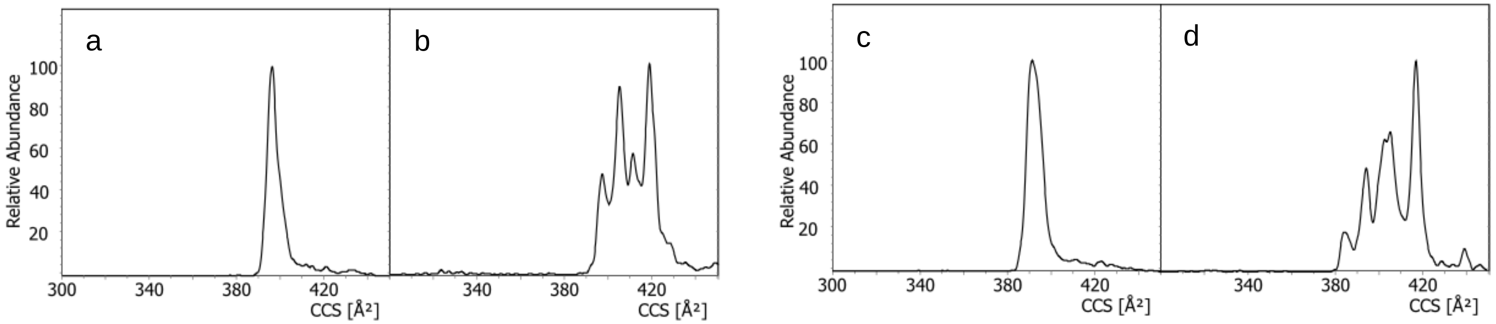

S5. Ion mobility patterns of Suc-Py (a) of 3B19 linear measured at a Δ6 value of 100.0 eV.

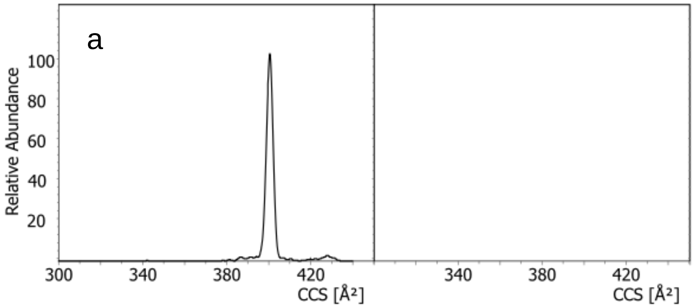

S6. Ion mobility patterns of Glu-IsoPy (a) and Glu-FeIsoPy (b) of 3C16 linear measured at a Δ6 value of 100.0 eV.

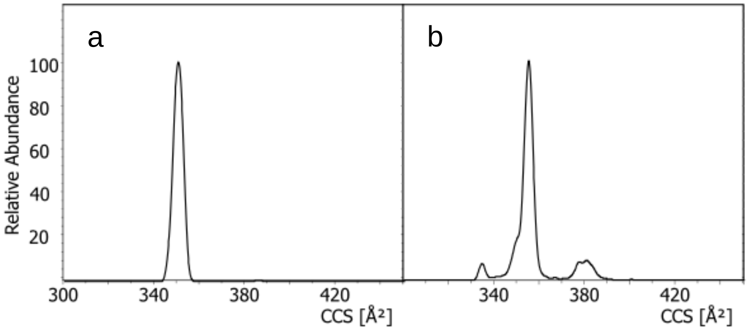

S7. Ion mobility patterns of Suc-Py (a), Suc-FePy (b), Suca-Py (c) and Suca-FePy (d) of 3D19 measured at a Δ6 value of 100.0 eV.

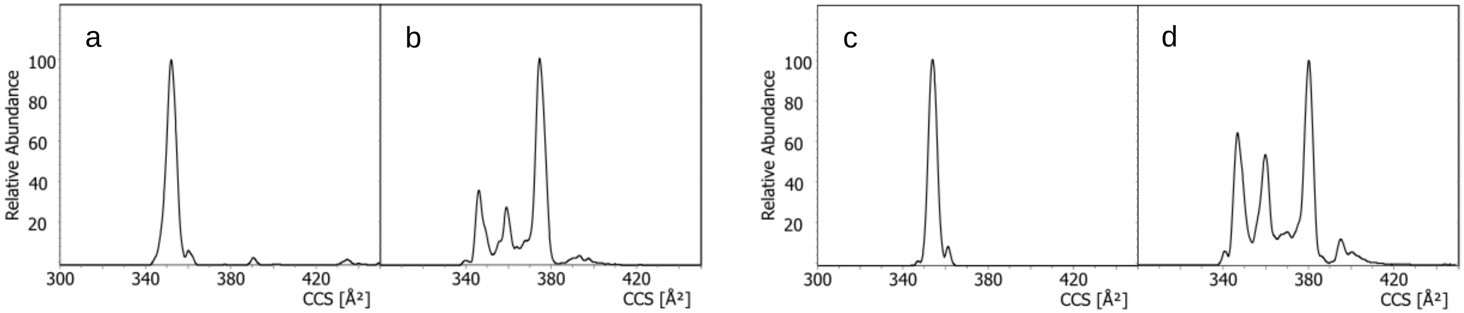

S8. Ion mobility patterns of Suc-Py (a), Suc-FePy (b), Suca-Py (c) and Suca-FePy (d) of 3F12 measured at a Δ6 value of 100.0 eV.

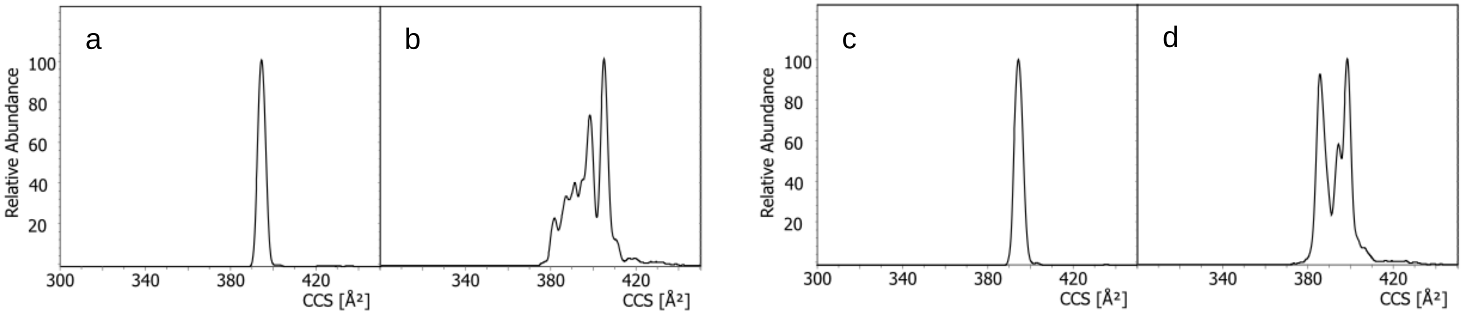

S9. Ion mobility patterns of Suc-Py (a), Suc-FePy (b), Suca-Py (c) and Suca-FePy (d) of 3G07 cyclized measured at a Δ6 value of 100.0 eV.

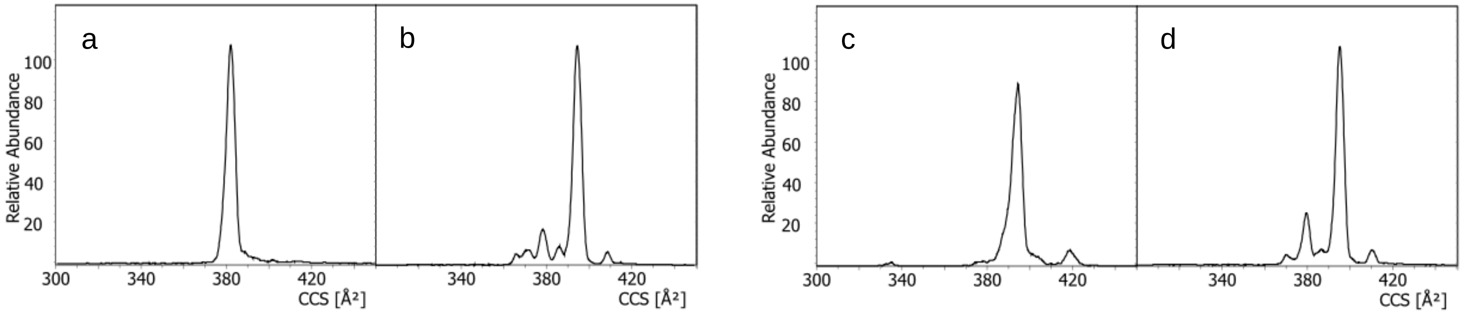

S10. Ion mobility patterns of Suc-Py (a) and Suc-FePy (b) of 3G07 linear measured at a Δ6 value of 100.0 eV.

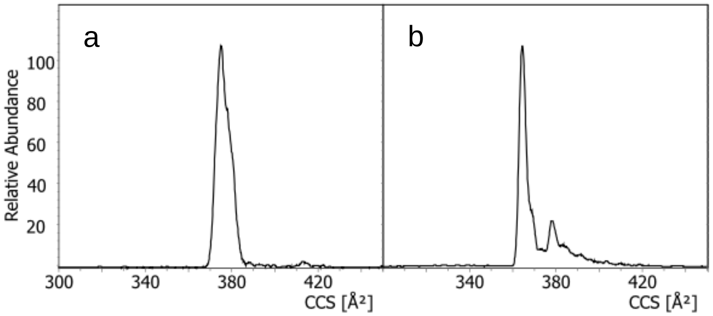

S11. Ion mobility patterns of Suc-Py (a), Suc-FePy (b), Suca-Py (c) and Suca-FePy (d) of PAO1 measured at a Δ6 value of 100.0 eV.

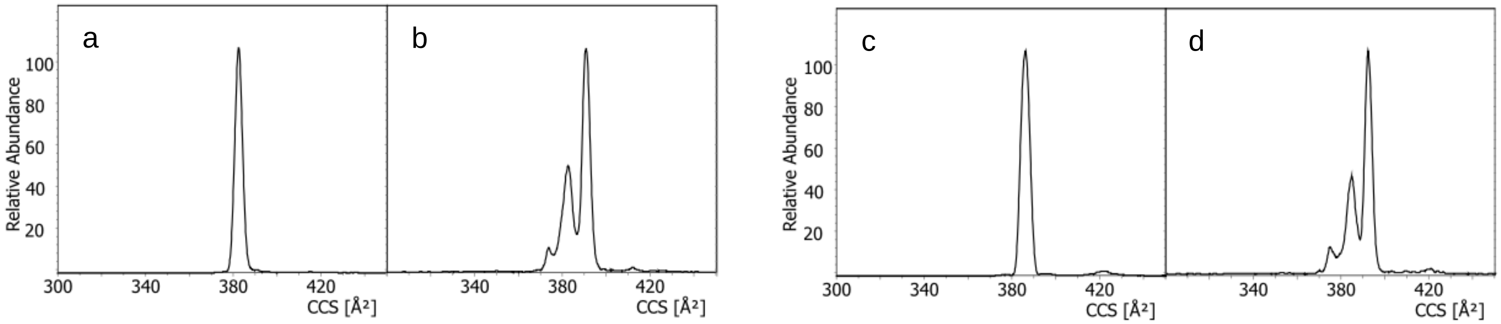

S12. Ion mobility patterns of Suc-Py (a), Suc-FePy (b), Suca-Py (c) and Suca-FePy (d) of Py SA measured at a Δ6 value of 100.0 eV.

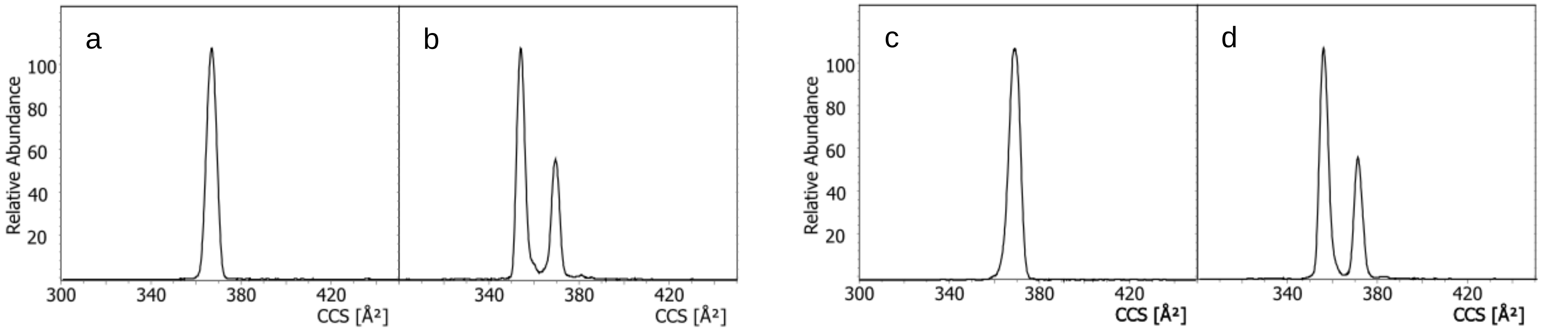

S13. Ion mobility patterns of Suc-Py (a), Suc-FePy (b), Suca-Py (c) and Suca-FePy (d) of S3a05 measured at a Δ6 value of 100.0 eV.

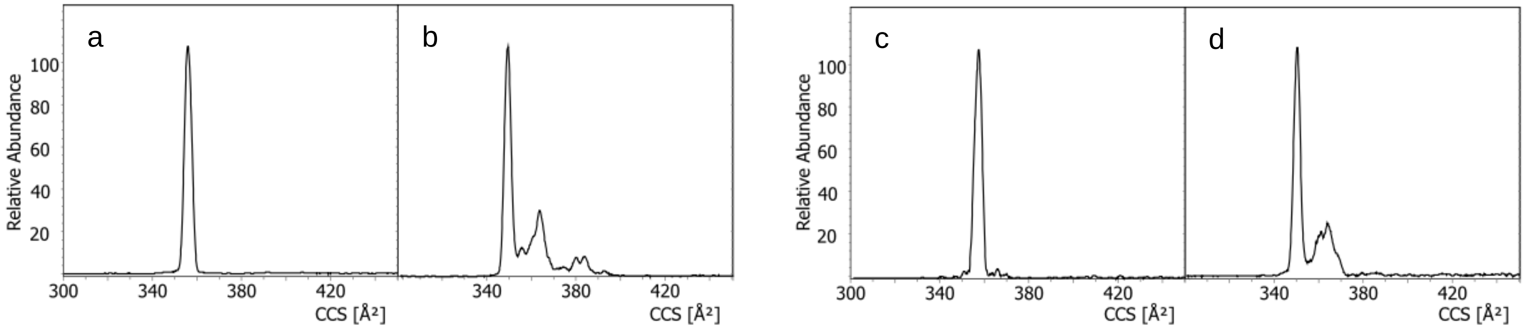

S14. Ion mobility patterns of Suc-Py (a), Suc-FePy (b), Suca-Py (c) and Suca-FePy (d) of S2a20 measured at a Δ6 value of 100.0 eV.

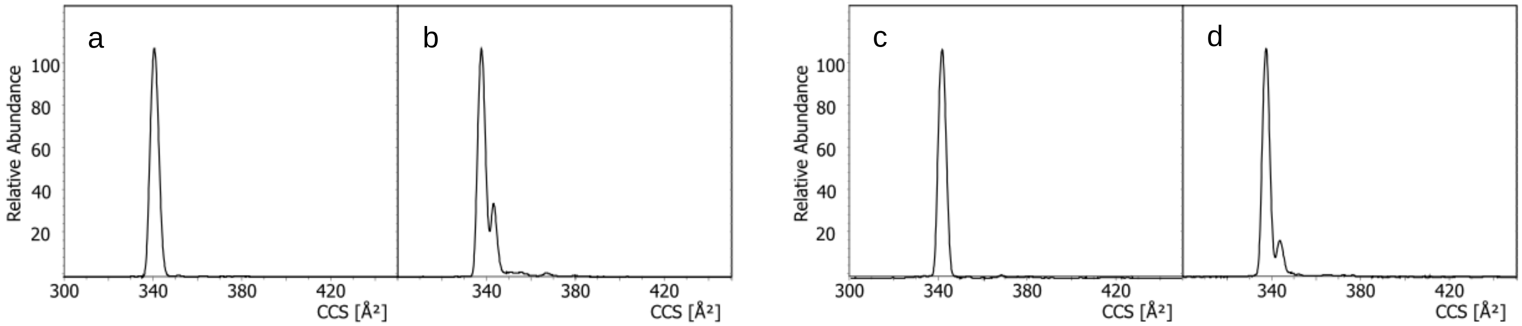

S15. Ion mobility patterns of Suc-Py (a), Suc-FePy (b), Suca-Py (c) and Suca-FePy (d) of S3b09 measured at a Δ6 value of 100.0 eV.

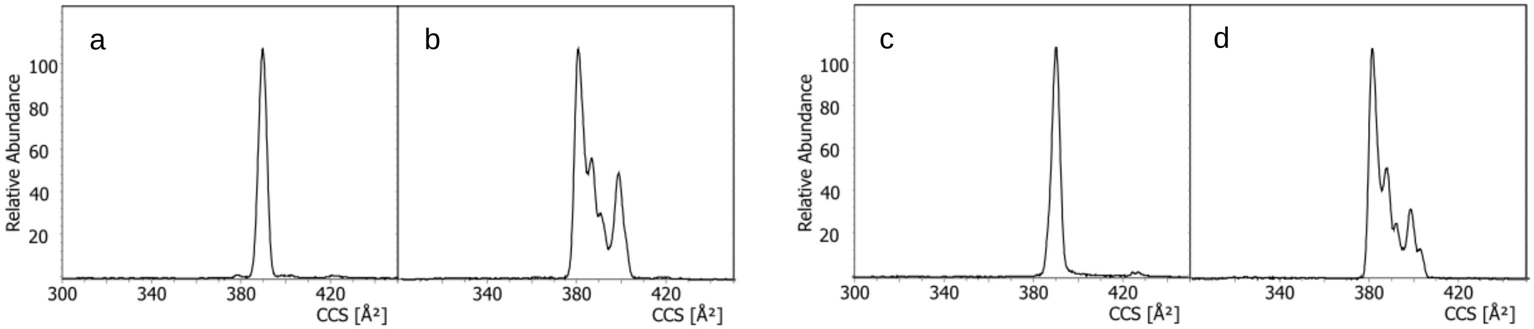

S16. Ion mobility patterns of Suc-Py (a), Suc-FePy (b), Suca-Py (c) and Suca-FePy (d) of S3b16 measured at a Δ6 value of 100.0 eV.

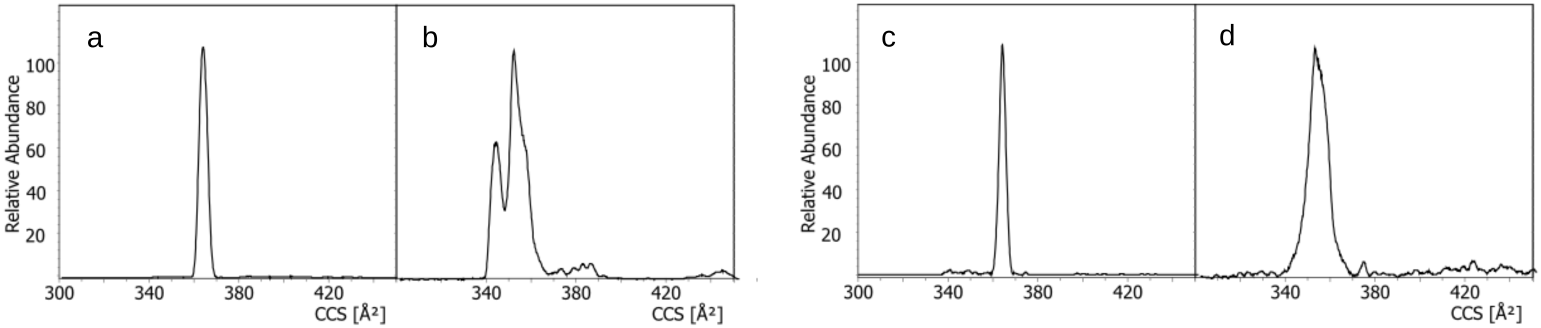

S17. Ion mobility patterns of Suc-Py (a), Suc-FePy (b), Suca-Py (c) and Suca-FePy (d) of S3c13 measured at a Δ6 value of 100.0 eV.

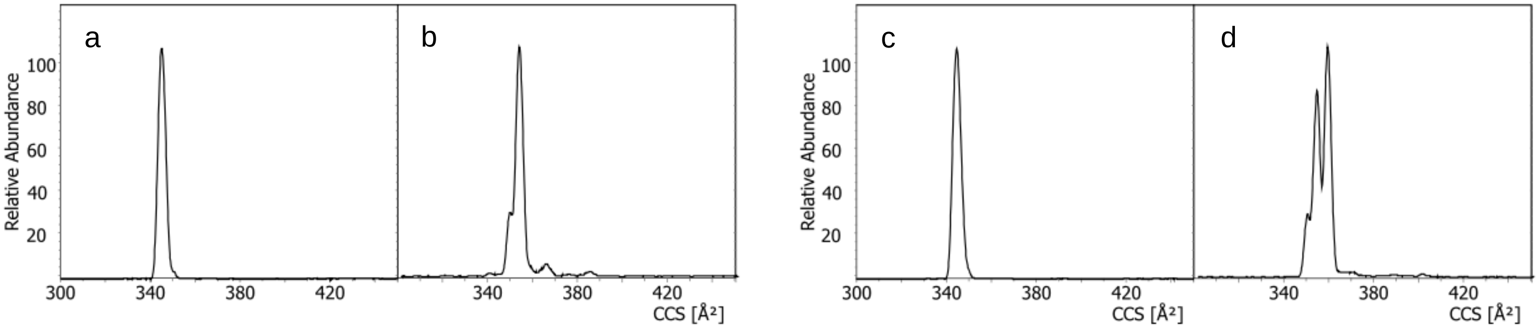

S18. Ion mobility patterns of Suc-Py (a), Suc-FePy (b), Suca-Py (c) and Suca-FePy (d) of S3e20 measured at a Δ6 value of 100.0 eV.

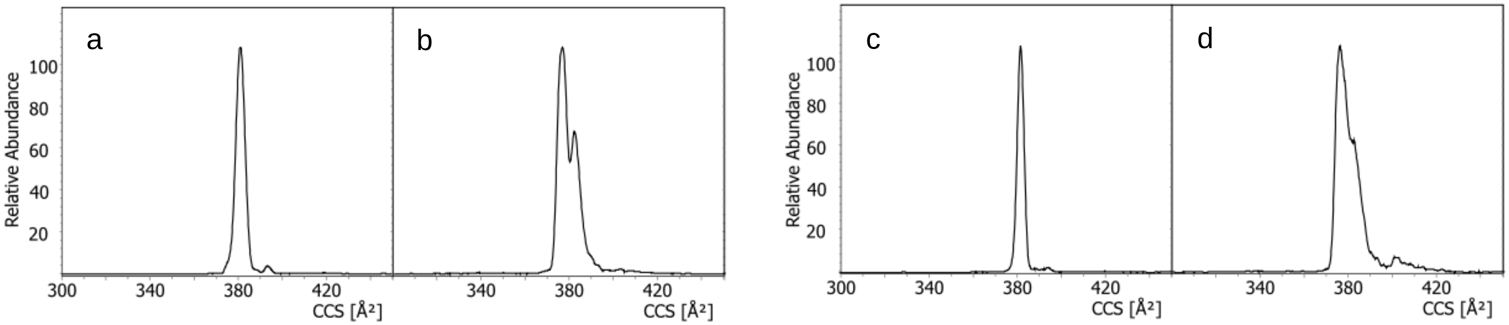

S19. Ion mobility patterns of Suc-Py (a), Suc-FePy (b), Suca-Py (c) and Suca-FePy (d) of S3g01 measured at a Δ6 value of 100.0 eV.

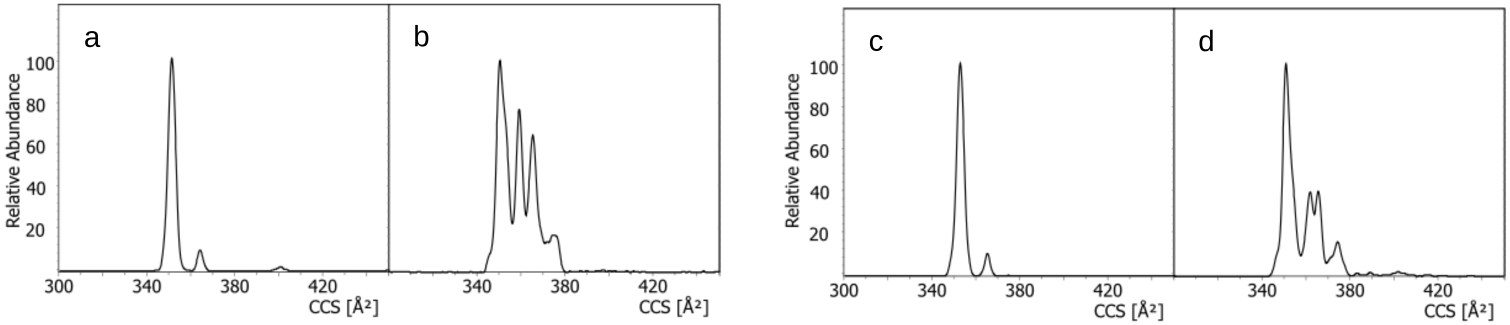

Supplement: Supplementary file 1 — Supplementary file1 (PDF 809 KB) [file 10534_2022_454_MOESM1_ESM.pdf]
